# Evaluating Circadian Dysfunction in Mouse Models of Alzheimer’s Disease: Where Do We Stand?

**DOI:** 10.3389/fnins.2020.00703

**Published:** 2020-07-07

**Authors:** Patrick W. Sheehan, Erik S. Musiek

**Affiliations:** ^1^Department of Neurology, Washington University School of Medicine in St. Louis, St. Louis, MO, United States; ^2^Knight Alzheimer’s Disease Research Center, Washington University School of Medicine in St. Louis, St. Louis, MO, United States

**Keywords:** circadian, Alzheimer’s disease, clock, tau, amyloid

## Abstract

Circadian dysfunction has been described in patients with symptomatic Alzheimer’s disease (AD), as well as in presymptomatic phases of the disease. Modeling this circadian dysfunction in mouse models would provide an optimal platform for understanding mechanisms and developing therapies. While numerous studies have examined behavioral circadian function, and in some cases clock gene oscillation, in mouse models of AD, the results are variable and inconsistent across models, ages, and conditions. Ultimately, circadian changes observed in APP/PS1 models are inconsistent across studies and do not always replicate circadian phenotypes observed in human AD. Other models, including the 3xTG mouse, tau transgenic lines, and the accelerated aging SAMP8 line, show circadian phenotypes more consistent with human AD, although the literature is either inconsistent or minimal. We summarize these data and provide some recommendations to improve and standardize future studies of circadian function in AD mouse models.

## Introduction

Numerous human studies have demonstrated that changes in circadian function are common in Alzheimer’s disease (AD) patients and contribute to disease morbidity. Circadian changes observed in AD patients include circadian fragmentation and decreased amplitude of circadian rhythms, which generally manifest as increased wakefulness at night and increased napping during the day (Satlin et al., 1995; Ancoli-Israel et al., 1997). These changes are observed in patients with preclinical AD pathology, meaning that they harbor amyloid plaque and/or tau pathology but do not yet have cognitive symptoms (Musiek et al., 2018). They are also evident in the mild cognitive impairment stage and worsen with disease severity. In symptomatic AD patients, a phase delay has been described, which means that the peak of activity occurs later in the day (Satlin et al., 1995; Ancoli-Israel et al., 1997). This phase delay has been hypothesized as a possible cause of “sundowning” in AD, or increased confusion and agitation in the afternoon and evening (Volicer et al., 2001). As the disease progresses, some patients develop severe fragmentation of circadian rhythms, leading to poor behavioral differentiation between day and night (Ancoli-Israel et al., 1997; Hatfield et al., 2004). The underlying mechanisms governing this circadian dysfunction are not well understood, creating a need for accurate mouse models which replicate some of these phenotypes.

Circadian biology can be a daunting field for AD researchers. Thus, a brief introduction to common circadian parameters may be helpful (Banks and Nolan, 2011). Circadian period is the length of time of a full circadian cycle. This can only be measured under “constant conditions” (usually meaning constant darkness for mice, indicated as “DD”). Most mice have a period just under 24 h in constant darkness; shortening or lengthening of period can be indicative of changes in the circadian clock itself. Amplitude indicates the difference between the peak and nadir of activity, averaged over several days. In general, mice should have minimal activity during the light phase (as they are nocturnal and sleeping), but are very active during the dark phase. Increased activity during the light phase and decreased activity in the dark would indicate blunted circadian amplitude, as is seen in AD patients. However, generally hyperactive animals can be overactive during the dark phase and have a high amplitude, while hypoactive animals may be inactive during both phases and show artificially blunted amplitude. If these differences in baseline activity levels are altered in transgenic mouse models, it may lead to difficulty interpreting circadian behavioral analyses. Phase indicates the time of day of peak activity, averaged over several days. A later peak (phase delay) is seen in AD patients. Fragmentation indicates a breakdown of circadian timing to drive consolidated periods of rest and activity. Fragmentation usually leads to a blunted amplitude, or can be measured by a non-parametric test called intradaily variability (IV) (Huang et al., 2002). Of note, circadian rhythms in behavior are largely driven by the activity of the suprachiasmatic nucleus (SCN) of the hypothalamus, the “master clock” of the body. Degeneration or dysfunction of the SCN, as has been described in human AD (Swaab et al., 1985), can lead to weaker circadian synchronization, increased fragmentation, and decreased amplitude (Nakamura et al., 2011). At a molecular level, circadian rhythms are driven by oscillations of circadian clock genes in the SCN and other tissues. These circadian clock genes include *Bmal1* and *Clock*, which function as transcription factors to drive transcription of their own repressors: *Per1, Per2, Per3, Cry1, Cry2*, and Rev-erbα and β. Levels of these clock gene transcripts oscillate with a 24-h period in most tissue and are entrained to daily light cycles (Buhr and Takahashi, 2013). Thus, measurement of clock gene oscillations in SCN and other tissues can be a molecular marker of clock function.

Over the past 20 years, a considerable literature has arisen in which circadian parameters have been examined in various AD models. Many of these studies have described modest changes in AD model mice, though these changes vary considerably between studies. The sheer number of distinct AD mouse models, as well as the age- and sex-dependency of pathology in these models, has complicated things further. Thus, it remains difficult to identify AD models which consistently and faithfully recapitulate the findings observed in human AD. Below, we have attempted to divide AD mouse models into their most common categories, and to review the existing literature on circadian rhythms in these models. These data are compiled in Table 1. While not an exhaustive or systematic review, the included studies represent many of the vast majority of published studies, as well as the most commonly cited and most thorough studies of circadian function in AD mouse models (other species were excluded), and thus provide what we believe is a representative sample of the literature.

**TABLE 1:** Summary of studies of circadian function in mouse models of AD.

Citation/PMID	Mouse model	Age	Conclusions/effects observed	DD	Comments
Oyegbami et al., 2017 28317486	APPswe/PS1dE9	2 months	Transgenic (Tg) mice have increased daily activity and increased activity amplitude with a slightly shorter period. *Cry1* and *Cry2* expression seems blunted in the medulla pons at ZT2 and ZT14	Yes	No pathology shown. Measurements are taken before pathology should be present. Difficult to tell whether oscillations in clock genes are blunted with only two time points
Baño Otalora et al., 2012 22823866	APPswe/PS1dE9	3.5–5.5 months	Response to phase shifting paradigms was not altered in Tg mice. Body temperature increased in the light phase in Tg mice and intradaily variability of body temperature was not affected. Tg mice showed no difference in period or locomotor activity	Yes	No pathology shown. Measurements are taken before pathology should be present
Kent et al., 2019 30884411	APPswe/PS1dE9	6, 9, 12, and 19 months	No difference in period, response to phase shifting, total daily activity, melanopsin expression, ultradian rhythms, intradaily variability, number or duration of activity bouts, food anticipatory behavior, synchrony in peripheral oscillators or total time spent asleep. Tg mice have slightly delayed activity onset and exhibit increased activity in the second half of the night	Yes	Pathology was only shown at 7 and 10 months of age and was not correlated to circadian parameters assessed
Ma et al., 2016 27796320	APPswe/PS1dE9	12–15 months	*Per2* and *Cry1* mRNA amplitudes decreased in hippocampus in Tg mice sampled at 6-h time intervals	No	mRNA rhythms were not quantified. Age is appropriate to have plaque pathology
Paul et al., 2018 29540298	tg-SwDI (APP mutant)	3, 6, 10 months	Tg mice with shortened period in DD, more variable activity onset/offset. SCN neuronal firing amplitude decreased (less during day, more during night)	Yes	SCN electrical records are unique. No pathology shown
Ni et al., 2019 31470863	APP-KI	2 months	Isolated cortical microglia from 2-month-old Tg mice at 4-h time intervals have less *Bmal1*, *Per2*, and *Rev-erbα* on average. Amplitude of most clock genes were decreased in these microglia	No	Did not show pathology. Mice this young should not have disease pathology
Bedrosian et al., 2011 21709248	Tg2576 (APPswe)	5 and 9 months	Increased daily activity in the dark phase in Tg mice	No	No constant conditions or pathology shown
Wisor et al., 2005 15708480	tg2576 (APPswe)	5–17 months	Age-dependent increase in period in DD. No other circadian analyses	Yes	No pathology shown
Sundaram et al., 2019 31551449	APPSwe crossed with PS1 line 5.1	9–10 months	Tg mice have a slightly shorter period in DD and increased intradaily variability. No difference in overall activity observed but Tg mice had an increased activity amplitude at the peak of the active cycle which did not persist in DD	Yes	
Duncan et al., 2012 22634208	APP^*NLH*^/PS-1^*P264L*^	4, 11, and 15 months	Tg mice show no difference in activity rhythms. VIP and vasopressin were not altered in the SCN. No change in AM/PM *Per2* expression in the hippocampus, cingulate or motor cortex, but blunted PM *Per2* expression in SCN of Tg mice. Amount of wheel running activity in the light phase was significantly decreased in Tg mice	No	Showed pathology at all time points using an Aβ ELISA. No constant conditions
Lee et al., 2020 31800167	5x FAD (APP/PS1)	6.5 months	BMAL1 protein is decreased in the cortex of Tg mice. *Per2* mRNA is decreased in the hippocampus and cortex of Tg mice while *Per1* is only shown to decrease in the hippocampus	No	Only checked expression level at a single time point throughout the day
Song et al., 2015 25888034	5x FAD (APP/PS1)	2 and 8 months	Tg mice have decreased activity at 8 months of age and decreased body temperature amplitude that persists in DD. Protein levels and mRNA expression of *Bmal1* and *Rev-erbα* are altered in the SCN of 2-month-old Tg mice measured at 4-h intervals. BMAL1 protein rhythms are blunted	Yes	No rhythmic analysis on mRNA or protein measurements. No pathology shown. mRNA analysis was done before pathology should be present
Boggs et al., 2017 28958954	J20 APP/Apoe4	6 and 12 months	Activity onset was delayed in Tg mice at 6 and 12 months of age. Tg mice show decreased activity in the light phase at 12 months of age and no difference in activity in the dark phase	No	No pathology shown
Ambrée et al., 2006 15993515	TgCRND8	30, 60, 90, 120 days	Increased daily activity in Tg mice at all ages	No	No constant conditions and no pathology shown. Circadian changes occur before pathology is likely present
Adler et al., 2019 31334659	3xTG-AD	10–11 months	Tg mice have a slightly shorter period in DD and displayed irregular activity onsets	Yes	No pathology shown
Knight et al., 2013 22864021	3xTG-AD	4, 6, 8, 9, 10 months	Tg mice have a higher body temperature amplitude as they age. Increase in activity amplitude not seen until mice are 10 months of age. No pathology seen in the hypothalamus of 12-month-old Tg mice	No	No constant conditions and pathology is only shown at one time point (12 months old)
Wu et al., 2018 29626648	3xTG-AD	6 months	Tg mice have a slightly shorter period with a lower daily activity and a smaller activity amplitude. Tg mice also have an increase in intradaily variability. *Per1* and P*er2* mRNA in the SCN seem to have a phase delay of 4 h in Tg mice	Yes	Never show differences in pathology, no statistical analysis of rhythms or phase. mRNA not harvested under DD
Bellanti et al., 2017 28671110	3xTG-AD	6 and 18 month old	Examine mRNA of several clock genes in SCN, hippocampus, and frontal cortex of 3xTG mice, at lights on (ZT0, 7 am) or lights off (ZT12, 7 pm). Two ages assessed. Blunting of Bmal1 expression in the SCN, along with some other changes in clock gene expression in older mice	No	No constant conditions, cannot differentiate effects of light exposure from those of circadian time. Multiple brain regions and ages is a strength
Sterniczuk et al., 2010 20471965	3xTG-AD	Various	Tg male mice have elevated light and dark phase activity, not age-dependent. Female Tg mice post-plaque pathology show decreased activity during dark. Shorter period in males only in DD. Male Tg mice have fewer AVP and VIP cells in the SCN (females not assessed). No difference observed in response to phase shifting	Yes	Only period is assessed in DD. No pathology shown outside of SCN, although SCN pathology and sex discrimination is a strength
Stevanovic et al., 2017 28461004	Tg4510 Tau	8 months	Tg mice have a longer free running period and are more active in the light phase, which does not persist in constant conditions. PER2 protein is decreased in the hypothalamus and hippocampus of Tg mice at two time points, with no difference in BMAL1 expression. Phosphorylated tau is present in the SCN at 8 months of age	Yes	Only checked at one age-unclear if these effects are due to the progression of pathology. Harvest of tissue in DD is a strength. Difficult to make conclusions of Bmal1 and Per2 oscillations with only two time points
Miyamoto et al., 1986 3786521	SAMP8	2,6,8,12 months	Tg mice have increased activity in the light phase and decreased in dark phase, decreased amplitude	No	No constant conditions
Pang et al., 2004 14675737	SAMP8	2, 7, 12 months	Period of SAMP8 mice does not change with age, although overall activity declines	Yes	Compared Tg mice to themselves at various ages, not to WT mice
McAuley et al., 2002 12009511	SAMP8	2, 7, 12 months	SAMP8 mice show decreased amplitude, increases activity during light phase, and rhythm “splitting” with additional peak of activity	Yes	Compared Tg mice to themselves at various ages, not to WT mice
Sánchez-Barceló et al., 1997 9383106	SAMP8	4-5 months	SAMP8 mice show no difference in vasopressin staining in the SCN at ∼5 months of age. They also entrain faster to a 6-h phase shift	N/A	
Zhou et al., 2016 7824104	Apoe^–/–^	6 weeks	KO mice show increased onset variability, decreased activity amplitude in DD, slower entrainment to light shift. Increased clock gene expression variability in SCN, increased tau aggregation and synaptic degeneration in SCN, and decreased melanopsin in retina	Yes	Not a true AD model, relevance of ApoE KO to AD is unclear
Wang et al., 2016 27021954	Aβ_31–35_ injection into hippocampus of WT mice	6–8 weeks	Injected mice had lengthened period in DD, blunted clock gene expression in SCN and heart	Yes	Unusual AD model, relevance to AD pathogenesis is unclear
Plucinska et al., 2014 25100603	PLB4 mice (hBACE1 knockin)	3, 6, 12 months	No differences noted in circadian rhythms, except for a significant decrease in activity in both the light and dark phase in Tg mice at 6 months of age. This measurement was trending but not significant at 12 months	No	Model avoids APP overexpression. Pathology is shown. Mouse develops intracellular amyloid rather than plaques, relevance is unclear

### APP and APP/PS1 Mice

There are a multitude of different transgenic mouse lines which express human amyloid precursor protein (APP) and in some cases human presenilin 1 (PS1), both harboring a variety of AD-associated mutations which promote amyloid-beta (Aβ) generation and aggregation. These mice accumulate amyloid plaque pathology at variable ages and in varying brain regions depending on the specific transgene and sex of the animal. APP/PS1 mice also vary in the biochemical character of the plaques that are formed, as well as the presence or absence of intracellular amyloid. Presumably, APP and APP/PS1 mice model the earliest stage of AD, that being amyloid plaque deposition, as most of these models develop minimal tau pathology or overt neurodegeneration. Humans with preclinical AD or MCI have been shown to have decreased circadian amplitude, increased fragmentation, and, in MCI, a phase delay (Naismith et al., 2014; Ortiz-Tudela et al., 2014). However, results of circadian studies in APP and APP/PS1 mice do not clearly demonstrate consistent AD-like phenotypes. Several studies have shown increased activity in these mouse lines during the dark phase (the active phase of mice), as well as increases in circadian amplitude (Ambrée et al., 2006; Bedrosian et al., 2011; Baño Otalora et al., 2012; Oyegbami et al., 2017). Aside from this, the wide variety of subtle and oft-conflicting circadian phenotypes in APP and APP/PS1 mouse studies can be appreciated in Table 1. Thus, a clear circadian phenotype which models early human AD has not emerged from the literature. Recently, Kent et al. conducted a very thorough study of APPswe/PS1dE9 mice, a commonly used APP/PS1 model which developed plaques around 6 months old (mo). This study, which examined mice at multiple ages and included a variety of light manipulations and *Per2*-luciferase rhythm recordings in SCN and various peripheral tissues (such as liver, lung, and spleen), revealed only a minimal delay in activity onset after lights-off in transgenic mice (Kent et al., 2019). Moreover, several studies demonstrate circadian changes which precede amyloid plaque pathology, suggesting possible strain or transgene effects, though an effect of soluble Aβ cannot be excluded (Bedrosian et al., 2011; Ortiz-Tudela et al., 2014; Oyegbami et al., 2017; Ni et al., 2019). While not a true circadian study, Roh et al. (2012) demonstrated blunting of amplitude in diurnal rhythms in sleep and brain lactate levels in APPswe/PS1dE9 mice, which was reversible with immunization of an anti-Aβ antibody, suggesting that amyloid pathology may indeed contribute to some aspects of rhythm dysfunction. Paul et al. (2018) examined the TgSwDI APP mutant mouse and observed shortened period and increased variability in activity onset/offset that were associated with blunted amplitude of neuronal firing in the SCN, providing an electrophysiological basis for rhythm disturbance. A human BACE1 knockin mouse, which drives intracellular amyloid accumulation without APP overexpression, showed decreased dark-phase activity but no other major circadian alterations, though assessment was limited (Plucinska et al., 2014). An issue with the wide array of APP and APP/PS1 is that these transgenes are driven by specific promoters (including the Thy1 and prion protein promoters) which may not express in all relevant cell types and regions (such as the SCN and circadian output pathways). This variability of transgene expression may account for some of the inconsistency in circadian phenotypes across APP/PS1 models. While APP and APP/PS1 models have been the most studied in terms of circadian function, the wide array of unique APP and APP/PS1 lines, as well as the possible amyloid-independent effects of these transgenes on activity level, has led to a murky literature which undermines the utility of these models for examining circadian changes.

### Tauopathy Models

Tau transgenic mice overexpress the human MAPT gene with disease-causing mutations (often P301S or P301L) and are often used to model tauopathy associated with AD. Unlike APP mice, tau transgenics generally develop striking neurodegeneration and premature death. The Tg4510 mouse, an aggressive model of tauopathy which expresses MAPT^*P301L*^ (Ramsden et al., 2005), develops a lengthened circadian period and decreased circadian amplitude, as well as seemingly blunted circadian clock gene expression in the hypothalamus and hippocampus when measured at two times of day (Stevanovic et al., 2017). These changes were observed at 8 mo, when severe tau pathology is present. SCN tau pathology was also noted in this study. A study of MAPT^*P301S*^ PS19 mice, which focused exclusively on sleep, showed a decrease in sleep and an increase in wakefulness, suggesting a possible underlying circadian deficit, very late in the disease progression (Holth et al., 2017). While promising, further studies of circadian function in tauopathy models are needed to determine how robust and consistent these changes are across tau models and studies.

### 3xTG Mice

The 3xTG mouse was introduced in 2003 as a murine model of both of the hallmark neuropathologies of human AD: amyloid plaques and tau tangles. 3xTG mice express three transgenes (APP Swedish, MAPT P301L, and PS1 M146V) and develop amyloid plaque pathology starting around 4 months, with aggregated tau pathology beginning around 12 months (Oddo et al., 2003). Several studies have examined circadian function in 3xTG mice, some of which describe phenotypes which more closely resemble human AD. However, results are fraught with inconsistency across different studies. Wu et al. (2018) show a striking decrease in amplitude, less daily activity, and fragmentation (increased IV) in 6 mo 3xTG mice, reminiscent of human AD circadian dysfunction. Knight et al. (2013) report no changes in activity profile in 6 mo 3xTG mice and go on to show an increased amplitude in daily activity and temperature rhythm by 10 months. Moreover, Sterniczuk et al. (2010) show increased amplitude and daytime activity in four month old male mice while female mice show a decreased amplitude by 11 months. Finally, Adler et al. (2019) showed a shortened period and decreased amplitude in 3xTG mice which was ameliorated by inhibiting casein kinase 1δ and 1ε, enzymes that are important for the degradation of PER proteins and maintenance of core clock function. Thus, while the 3xTG mouse shows some promise as a more accurate model of AD-like circadian changes, the inconsistency across studies makes interpretation difficult. It is also notable that these circadian changes were all observed at ages prior to the accumulation of considerable tau pathology. Of note, a different triple-transgenic mouse expressing APP, hTau, and PS1, specifically in the forebrain (PLB1 mice), did not show obvious circadian deficits, suggesting that SCN-dependent expression of pathology may be important (Platt et al., 2011).

### Other AD Models

The Senescence Accelerated Mouse, line P8 (SAMP8 mouse) is a unique, non-transgenic mouse line that has been selectively bred to promote accelerated aging (Butterfield and Poon, 2005; Ramsden et al., 2005; Holth et al., 2017; Stevanovic et al., 2017). SAMP8 mice are usually compared to another SAM mouse line which is aging-resistant (SAMR1) as a control. SAMP8 mice spontaneously develop mild amyloid-beta accumulation, mild tauopathy, synapse loss, and cognitive impairment early in life, and have been used as a model of AD (Morley et al., 2012). SAMP8 mice show striking circadian changes, including increased activity during light phase, decreased circadian amplitude, and fragmentation (Miyamoto et al., 1986; McAuley et al., 2002; Pang et al., 2004), although this has not always been reported (Sánchez-Barceló et al., 1997). Like APP mice, they show a general hyperactivity phenotype, which complicates interpretation. However, the striking circadian changes observed in several studies suggest that SAMP8 mice have potential as a model of age and AD related circadian dysfunction.

Apolipoprotein E (ApoE) genotype is the major genetic risk factor for sporadic AD, with the E4 allele imparting increased risk. Apoe−/− mice have been reported to have more variable activity rhythms in DD, impaired entrainment, and alterations of SCN clock gene expression rhythms (Zhou et al., 2016). However, APP mice expressing human ApoE4 do not have more severe circadian changes than APP with wild type mouse ApoE (Graybeal et al., 2015; Boggs et al., 2017). This is an unexpected finding, as ApoE4 increases amyloid pathology in mice (Castellano et al., 2011) and might be expected to exacerbate circadian dysfunction. More detailed studies which incorporate pathology assessments are needed. Injection of Aβ peptide into the brain of wild type mice has also been used to model AD, and has been reported to lengthen period and blunt clock gene rhythms in the SCN (Navigatore-Fonzo et al., 2017), and to alter expression patterns of *Apoe* and other mRNAs in the hippocampus (Wang et al., 2016), though this model is not widely used.

### Impact of AD Pathology on Clock Gene Expression

Differences in circadian outputs at the level of sleep-wake cycles can generally be correlated to changes in the molecular clock. However, differences in clock gene expression in studies of AD models are generally difficult to interpret. For example, some studies have suggested decreased expression of clock genes in AD models before the onset of pathology. Oyegbami et al. (2017) show a decrease in *Cry1* and *Cry2* in the medulla pons of 2-month-old APPswe/PS1dE9 mice, while Ni et al. (2019) show a decrease in *Bmal1*, *Per2*, and *Rev-erbα* in isolated cortical microglia from 2-month-old APP-KI mice (both well before plaque deposition occurs). Song et al. (2015) showed blunting of rhythmic BMAL1 protein levels prior to disease pathology in the SCN of 2 mo 5xFAD mice (an APP/PS1 mutant model). These may indicate an effect of soluble Aβ, or effects of the transgene/mutation introduced, highlighting the importance of these assays at multiple ages over the course of disease pathology. Wu et al. (2018) showed a slight phase delay and decreased expression of *Per1/2* in the SCN of 3xTG mice, while Ma et al. (2016) showed altered rhythms in hippocampal *Bace2* and *Apoe* mRNA. Other studies in AD models have examined clock gene expression at one or two time points throughout the circadian cycle, usually early morning and early evening, in some case showing a loss of variation in AD mice (Duncan et al., 2012; Bellanti et al., 2017; Stevanovic et al., 2017; Lee et al., 2020). While these can be used to make general conclusions, it is difficult to interpret if clock genes are changing amplitude, or if there is just a difference in phase of expression of that particular gene. Detailed time courses of circadian gene expression in different brain regions of AD mouse models at pathology-bearing ages are needed to thoroughly address this issue.

### Recommendations for Circadian Studies in AD Mouse Models

Considering the complexity of this literature, we offer here some recommendations for future studies of circadian rhythms in AD mouse models:

*Constant conditions:* Mouse activity must be recorded under constant conditions (usually constant darkness or dim light) in order to make firm conclusions about the circadian system. Many studies of behavioral rhythms in AD model mice are conducted in 12:12 h light:dark, which introduces factors related to light intensity, as well as the issue of “masking,” or behavioral suppression by light which occurs independently of the circadian system. Furthermore, previous studies have shown some rhythm abnormalities in AD models in 12:12 h light:dark cycles that resolve in constant conditions (Stevanovic et al., 2017; Sundaram et al., 2019). Moreover, tissue harvests for clock gene expression studies should also be conducted in DD, as light can impact clock gene expression (Moriya et al., 2000).

*Correlation with pathology:* Many studies of circadian function in AD models do not describe or quantify the degree of pathology in their mice. It is important to demonstrate the degree of pathology (amyloid plaque burden, tau pathology, etc.) in the mice at the same age and in the same sex that are used for behavioral studies. While previous papers can be used as general guidelines, mice of a similar genotype can have very different pathology when raised in different colonies and facilities. Moreover, circadian deficits arising in AD mice well before pathology may suggest behavioral effects of transgene overexpression, rather than true pathology-driven changes. Indeed, behavioral changes in 3xTG can be dependent on genetic background (Pardossi-Piquard et al., 2016). Finally, it is potentially important to assess the degree of pathology in the SCN in these mouse models, to see if there is a direct effect on the central pacemaker. SCN pathology has only been analyzed in a few models, as 3xTG mice do not have plaques or tangles at 12 months, while Tg4510 mice do have SCN tau pathology at 8 months (Knight et al., 2013; Stevanovic et al., 2017). Even in the absence of clear plaque pathology, it is possible that soluble Aβ species (such as oligomers) may impact SCN function, though levels and dynamics of soluble Aβ species in the SCN have not been examined. Finally, human post-mortem AD SCN generally shows neuronal loss and tau pathology, rather than plaques, which may not be recapitulated in amyloid-based models (Stopa et al., 1999).

*Multiple endpoints:* Most studies focus on rhythms in wheel running as the primary endpoint. While some have included temperature or some limited clock gene investigation, little is known about other circadian endpoints. Peripheral clocks, for instance, could be disrupted even in the face of normal-appearing wheel running behavior. Some studies do examine *Per2*-luciferase rhythms in SCN explants (Kent et al., 2019), clock gene expression in SCN (Song et al., 2015; Stevanovic et al., 2017), or SCN firing rate (Paul et al., 2018). Integration of multiple endpoints in future studies may reveal important phenotypes.

*Sex and age considerations:* Sex has a strong effect on both circadian rhythms, as well as on pathology in AD models. In general, female APP and APP/PS1 mouse lines develop amyloid plaques faster than males, while this relationship is reversed for the accumulation of tau pathology in MAPT mice (Carroll et al., 2010; Sun et al., 2020). Furthermore, there is potentially conflicting effects of sex on circadian biology that could further complicate these studies (Bailey and Silver, 2014). Thus, sex must always be considered. Age also has strong effects on circadian function, but is less clearly tied to amyloid/tau pathology in mouse models, as specific transgene/mutations can drive pathology at vastly different ages. In some cases, mice develop plaques when they are still young, while in others age and amyloid pathology come together and may interact (Wisor et al., 2005). Thus, the effects of age itself, and the rate of pathology accumulation, must be considered.

## Conclusion

In summary, while many AD mouse models exhibit alterations in circadian behavioral rhythms and/or gene expression, these changes are generally not consistent across studies or models, or have questionable relevance to human AD. As such, it is still unclear if specific protein pathologies (such as amyloid or tau aggregation) directly drive circadian changes, or if these observed changes are due to other factors (such as transgene overexpression, genetic background, sex, or age). As new AD models are developed, circadian studies must consider basic study design principles, degree of pathology, age, and sex, in order to provide interpretable and consistent results.

## Author Contributions

PS and EM assessed the literature and wrote the manuscript. PS created Table 1. PS and EM edited the manuscript. Both authors contributed to the article and approved the submitted version.

## Conflict of Interest

The authors declare that the research was conducted in the absence of any commercial or financial relationships that could be construed as a potential conflict of interest.

## Abbreviations

A β: amyloid-beta peptide
AD: Alzheimer’s disease
ApoE: apolipoprotein E
APP: amyloid precursor protein
DD: constant darkness
LD: 12 h light:dark cycle
IV: intradaily variability
PS1: presenilin 1
SAMP8: senescence accelerated mouse P8
SCN: suprachiasmatic nucleus
3xTG: APP/PS1/tau triple transgenic mouse.
